# Bartter-Like Syndrome as the Initial Presentation of Dent Disease 1: A Case Report

**DOI:** 10.3389/fped.2021.725251

**Published:** 2021-09-28

**Authors:** Qiaoping Chen, Yan Cao, Liyun Xu, Jingqi Liu, Xiaochuan Wu

**Affiliations:** Department of Pediatric Nephrology, Children's Medical Center, The Second Xiangya Hospital, Central South University, Changsha, China

**Keywords:** dent disease, bartter-like syndrome, low-molecular-weight proteinuria, hypercalciuria, *CLCN5* gene, children

## Abstract

Dent disease is a rare genetic disease characterized by low-molecular-weight proteinuria. Dent disease with Bartter-like syndrome is rare and can easily be misdiagnosed and mistreated. Herein, we report a case of Dent disease 1 with Bartter-like syndrome as the initial manifestation. The patient was admitted to The Second Xiangya Hospital of Central South University due to polydipsia, polyuria, and weakness of both lower limbs at 2 years of age. Laboratory tests showed that serum sodium, potassium and chlorine levels were low, while serum creatinine levels were normal. The calcium level in the urine was normal. The patient was initially diagnosed with Bartter syndrome, and despite medical interventions, he eventually developed chronic kidney disease stage 4 at 13 years of age. To determine the cause, the patient was recommended to undergo genetic testing, which showed a *CLCN5* gene c. 941C > T mutation (p.S314L), and was finally diagnosed as Dent disease 1. The clinical manifestations of Dent disease are complex and diverse. For patients with atypical clinical manifestations or unsatisfactory therapeutic effects, genetic testing is recommended.

## Introduction

Dent disease is an X-linked recessive renal tubular disorder characterized by low-molecular-weight proteinuria, with or without hypercalciuria, renal calcinosis, and progressive renal loss of renal function (1). In addition to low-molecular-weight proteinuria and hypercalciuria, Dent disease has some unusual presentations, such as Bartter-like syndrome and isolated proteinuria (2–5). Dent disease with Bartter-like syndrome is rare. To date, only 3 cases have been reported worldwide (2–4). The specific mechanism of Dent disease complicated with Bartter-like syndrome is not clear, some scholars have proposed that Dent patients with Bartter-like syndrome are more likely to progression to renal failure (4), while this hypothesis remains to be further confirmed.

Here, we report a case of Dent disease 1 with Bartter-like syndrome as the initial presentation.

## Case Report

The patient was a third child of unrelated parents and was born in February 2003. He had two sisters, both of whom were in good health. However, his grandmother has a history of uremia. At 2 years of age, the patient was admitted to The Second Xiangya Hospital of Central South University due to polydipsia, polyuria, and weakness of lower extremities. The 24-h urine output was about 4,000 ml, and the blood pressure was normal (90/50 mmHg). Laboratory tests showed normal serum creatinine level (57.8 umol/l), while low levels of serum sodium (110.4 mmol/l), potassium (2.02 mmol/l), chlorine (83.5 mmol/l) and phosphorus (0.45 mmol/l) were reported (Table 1). Serum calcium (2.38 mmol/l) and magnesium (1 mmol/l) were within normal values. The patient had metabolic acidosis (pH 7.321,HCO3-14.2mmol/l), but his urine calcium level was normal (0.067 mmol/kg·d). The physical examination of the eye and hearing was normal. He was diagnosed with Bartter syndrome, based on clinical manifestations and laboratory tests, and treatment began with potassium chloride sustained-release tablets, captopril, sodium bicarbonate, and calcitriol.

**Table 1:** Laboratory and urinary parameters in our patient.

**Age (years)**	**2**	**12**	**13**	**17**
pH	7.321	7.427		
HCO3-	14.2	28.2		
Serum Na (mmol/l)	110.4	141	134.6	141.4
Serum K (mmol/l)	2.02	2.84	3.94	4.44
Serum Cl (mmol/l)	83.5	99.6	94.4	104.5
Serum Ca (mmol/l)	2.38	2.42	2.3	
Serum Cr (μmol/l)	57.8	138	312.5	560.5
GFR [ml/ (min·1.73m^2^)]		44.72	24.95	16.67
Urine Protein	2+	2+	2+	1+
24 h Urine protein (mg/Kg)		23	13.7	16.6
β2 microglobulin (mg/l)	2.4		9.03	
24 h Urine calcium (mg/kg)	2.7			

Symptoms such as polydipsia and polyuria were significantly improved after treatment. The urine protein levels fluctuated from + to 2+, and serum potassium level was maintained at a normal low level. In order to control urinary protein, the patients were treated with prednisone and mycophenolate mofetil in 2015. However, despite medical interventions, the patient developed chronic kidney disease stage 4 at 13 years of age. To determine the pathological condition of the kidney, a renal biopsy was performed in 2016. Light microscopy of renal tissue showing focal segmental glomerulosclerosis and renal interstitial fibrosis. Electron microscopy of renal tissue showing mild glomerular lesion, no parabulbar organs, renal tubular epithelial edema and interstitial fibrous hyperplasia. Ultrasonography of our patient's in 2020 showed diffuse parenchymal lesions of both kidneys and multiple cysts in both kidneys.

Although the patient was initially diagnosed with Bartter syndrome, the symptoms were not typical. First, there was no obvious metabolic alkalosis and the patient had long-standing proteinuria. Second, most patients with Bartter syndrome have normal renal function and good prognosis (6), while our patient developed chronic kidney disease stage 4 at 13 years of age, which is relatively rare in Bartter syndrome. Third, the main pathological feature of Bartter syndrome is the proliferation and hypertrophy of para-glomerular organs (7) and the renal biopsy showed focal segmental glomerulosclerosis and interstitial fibrosis, which are rare in Bartter syndrome. Therefore, genetic testing was suggested and performed. The parents provide informed consent, and 2–3 ml of peripheral blood from both the child and parents were taken and used for genetic testing. The results showed a *CLCN5* gene c. 941C > T mutation (p.S314L), with the mother being a heterozygous carrier at this site. No mutation was detected in the pathogenic gene of Bartter syndrome. The patient was finally diagnosed with Dent disease 1.

## Discussion

According to the different mutant genes, Dent disease can be categorized into three types: Dent disease 1, Dent disease 2 and Dent disease 3. The mutation point of Dent disease 1 is located in the *CLCN5* gene encoding voltage-gated chloride channel protein 5 (ClC-5), while the mutation point of Dent disease 2 is located in the *OCRL* gene encoding an inositol polyphosphate 5-phosphatase, which is also the pathogenic gene of Lowe syndrome (8). In about 25% of the patients, no *CLCN5* and *OCRL* mutations were detected, and these patients were classified as Dent disease 3 (9). Chronic kidney disease occurs earlier in Dent disease 2 than in Dent disease 1 (10). Some studies have shown that the level of serum creatine kinase in patients with Dent disease 2 is significantly higher than that in patients with Dent disease 1 (11), which can be used to predict the genotype of patients with Dent disease.

Low-molecular-weight proteins can freely pass through the glomeruli and be reabsorbed in the renal tubules. Low-molecular-weight proteinuria is considered a sign of renal tubular dysfunction. Under the pathological condition, the reabsorption of low molecular weight protein in renal tubules decreases, resulting in low-molecular-weight proteinuria. Other hereditary renal tubular diseases, such as Lowe syndrome and Fanconi syndrome, can also have characteristic low-molecular-weight proteinuria. Lowe syndrome is caused by the *OCRL* gene mutation, and in addition to the manifestations of low-molecular-weight proteinuria, there are also other clinical symptoms such as congenital cataracts and developmental delay. In this case report, we wish to emphasize the importance of classifying the nature of proteinuria in patients with proteinuria. As low molecular weight proteinuria is considered an important feature of dent disease, it is recommended to perform genetic testing for patients with low molecular weight proteinuria, regardless of the presence or absence of other symptoms (12, 13).

Our patient had normal urine calcium levels at 2 years of age and no urine calcium test was done after that. A recent review described that hypercalciuria was absent in about 20% of Dent disease 1 and 15% of Dent disease 2 patients (14). Therefore, even proteinuria patients with normal urinary calcium cannot rule out the possibility of Dent disease. Meanwhile, it is important to consider the long-term monitoring of urinary calcium in such patients.

At first, our patient was diagnosed with Bartter syndrome and treated with potassium chloride sustained-release tablets, captopril, sodium bicarbonate, and calcitriol. Polydipsia and polyuria were significantly improved, serum potassium level was maintained at a normal low level. While the urine protein levels fluctuated from + to 2+, the patients were treated with prednisone and mycophenolate mofetil in 2015. At present, there is no effective cure for Dent disease. Clinically, it is mainly managed through symptomatic treatment support, with the purpose to control high urinary calcium and delay the disease progression. High-dose (> 0.4 mg/kg/d) thiazide diuretics can significantly reduce urinary calcium levels, however, there are also some side effects, such as severe dehydration and hypokalemia (15). A high potassium citrate diet has been shown to reduce urinary calcium and delay renal failure in animal experiments (16). There is no clear evidence that ACEI drugs reduce urinary protein in patients with Dent disease (17).

About 30% to 80% of patients with Dent disease develop chronic kidney disease at the age of 30–50 (18). While the patient in this case report developed chronic kidney disease stage 4 at the age of 13. There is an assumption that patients with Dent disease and Bartter-like syndrome symptoms are more likely to develop renal failure (4), however, this hypothesis needs more evidence to support it.

In conclusion, for patients with proteinuria whose clinical manifestations are not typical and therapeutic effects are not ideal, genetic testing and evaluating the dosage of low molecular weight urinary proteins are recommended to reduce unnecessary harm to patients.

## Data Availability Statement

The original contributions presented in the study are included in the article/supplementary material, further inquiries can be directed to the corresponding author/s.

## Ethics Statement

Written consent was obtained from the patient's family for the publication of the case report.

## Author Contributions

QC was the first-year resident and wrote the manuscript. YC was the attending physicians that participated in the care of the patient. LX and JL collected the patient's data. XW analyzed and interpreted the patient's data. All authors contributed to the article and approved the submitted version.

## Conflict of Interest

The authors declare that the research was conducted in the absence of any commercial or financial relationships that could be construed as a potential conflict of interest.

## Publisher's Note

All claims expressed in this article are solely those of the authors and do not necessarily represent those of their affiliated organizations, or those of the publisher, the editors and the reviewers. Any product that may be evaluated in this article, or claim that may be made by its manufacturer, is not guaranteed or endorsed by the publisher.

## References

[1] Mansour-Hendili L Blanchard A Le Pottier N Roncelin I Lourdel S Treard C. Mutation update of the CLCN5 gene responsible for dent disease 1. Hum Mutat. (2015) 36:743–52. 10.1002/humu.22804 25907713

[2] Besbas N Ozaltin F Jeck N Seyberth H Ludwig M. CLCN5 mutation (R347X) associated with hypokalaemic metabolic alkalosis in a Turkish child: an unusual presentation of Dent's disease. Nephrol Dialysis Transplant. (2005) 20:1476–9. 10.1093/ndt/gfh799 15814539

[3] Bogdanovic R Draaken M Toromanovic A Dordevic M Stajic N Ludwig M. A novel CLCN5 mutation in a boy with Bartter-like syndrome and partial growth hormone deficiency. Pediatr Nephrol. (2010) 25:2363–8. 10.1007/s00467-010-1615-x 20680351

[4] Okamoto T Tajima T Hirayama T Sasaki S. A patient with dent disease and features of bartter syndrome caused by a novel mutation of CLCN5. Eur J Pediatr. (2012) 171:401–4. 10.1007/s00431-011-1578-3 21932010

[5] Gungor T Eroglu FK Yazilitas F Gur G Cakici EK Ludwig M. A case of Type 1 dent disease presenting with isolated persistent proteinuria. Turk Pediatri Arsivi-Turk Arch Pediatr. (2020) 55:72–5. 10.5152/TurkPediatriArs.2018.6540 32231453 PMC7096570

[6] Rudin A. Bartter's syndrome. A review of 28 patients followed for 10 years. Acta Med Scand. (1988) 224:165–71. 3421146

[7] Bartter FC Pronove P Gill JR Maccardle RC. Hyperplasia of the juxtaglomerular complex with hyperaldosteronism and hypokalemic alkalosis. A new syndrome. Am J Med. (1962) 33:811–28. 10.1016/0002-9343(62)90214-0 13969763

[8] Hichri H Rendu J Monnier N Coutton C Dorseuil O Poussou RV. From lowe syndrome to dent disease: correlations between mutations of the OCRL1 gene and clinical and biochemical phenotypes. Hum Mutat. (2011) 32:379–88. 10.1002/humu.21391 21031565

[9] Anglani F D'Angelo A Bertizzolo LM Tosetto E Ceol M Cremasco D. Nephrolithiasis, kidney failure and bone disorders in Dent disease patients with and without CLCN5 mutations. Springerplus. (2015) 4:492. 10.1186/s40064-015-1294-y 26389017 PMC4571032

[10] Sakakibara N Nagano C Ishiko S Horinouchi T Yamamura T Minamikawa S. Comparison of clinical and genetic characteristics between Dent disease 1 and Dent disease 2. Pediatric Nephrol. (2020) 35:2319–26. 10.1007/s00467-020-04701-5 32683654

[11] Park E Choi HJ Lee JM Ahn YH Kang HG Choi YM. Muscle involvement in Dent disease 2. Pediatric Nephrol. (2014) 29:2127–32. 10.1007/s00467-014-2841-4 24912603

[12] Edvardsson VO Goldfarb DS Lieske JC Beara-Lasic L Anglani F Milliner DS. Hereditary causes of kidney stones and chronic kidney disease. Pediatr Nephrol. (2013) 28:1923–42. 10.1007/s00467-012-2329-z 23334384 PMC4138059

[13] Zaniew M Mizerska-Wasiak M Załuska-Leśniewska I Adamczyk P Kiliś-Pstrusińska K Haliński A. Dent disease in Poland: what we have learned so far? Int Urol Nephrol. (2017) 49:2005–17. 10.1007/s11255-017-1676-x 28815356

[14] Gianesello L Del Prete D Anglani F Calò LA. Genetics and phenotypic heterogeneity of Dent disease: the dark side of the moon. Hum Genet. (2021) 140:401–21. 10.1007/s00439-020-02219-2 32860533 PMC7889681

[15] Blanchard A Vargas-Poussou R Peyrard S Mogenet A Baudouin V Boudailliez B. Effect of hydrochlorothiazide on urinary calcium excretion in dent disease: an uncontrolled trial. Am J Kidney Dis. (2008) 52:1084–95. 10.1053/j.ajkd.2008.08.021 18976849

[16] Cebotaru V Kaul S Devuyst O Cai H Racusen L Guggino WB. High citrate diet delays progression of renal insufficiency in the ClC-5 knockout mouse model of Dent's disease. Kidney Int. (2005) 68:642–52. 10.1111/j.1523-1755.2005.00442.x 16014041

[17] Vaisbich MH Henriques LDS Igarashi T Sekine T Seki G Koch VH. The long-term use of enalapril and hydrochlorothiazide in two novel mutations patients with Dent's disease type 1. J Brasil Nefrol. (2012) 34:78–81. 10.1590/S0101-28002012000100013 22441187

[18] Devuyst O Thakker RV. Dent's disease. Orphanet J Rare Dis. (2010) 5:28. 10.1186/1750-1172-5-28 20946626 PMC2964617

